# Study protocol: Effectiveness of the maternal RSVpreF vaccine by virus type

**DOI:** 10.12688/wellcomeopenres.24371.1

**Published:** 2025-12-05

**Authors:** Anna Mensah, Rebecca Symes, Chengetai Mpamhanga, Nick Andrews, Lynne Ferguson, Rory Gunson, Katja Hoschler, Beatrix Kele, Wei Shen Lim, Jamie Lopez Bernal, Ross McQueenie, Chris Robertson, Tiina Talts, Heather Whitaker, Kimberly Marsh, Conall Watson, Maria Zambon, Thomas Williams

**Affiliations:** 1 United Kingdom Health Security Agency, 61 Colindale Ave, London, NW9 5EQ, UK; 2 Department of Child Life and Health, University of Edinburgh, Edinburgh, EH16 4TJ, UK; 3 West of Scotland Specialist Virology Centre, Glasgow, Scotland, UK; 4 Nottingham University Hospitals NHS Trust, Nottingham, England, UK; 5 Public Health Scotland, Glasgow, Scotland, UK; 6 Department of Mathematics and Statistics, University of Strathclyde, Glasgow, Scotland, G1 1XH, UK

**Keywords:** Respiratory syncytial virus, maternal vaccination, bronchiolitis, vaccine effectiveness

## Abstract

**Background:**

Respiratory syncytial virus (RSV) is a virus with two antigenic types, A and B, that cause significant morbidity and mortality in infants globally. A recently developed maternal vaccination based on the prefusion F protein (“RSVpreF”) could have a significant impact on disease burden, if introduced globally. Whether or not the effectiveness of this vaccine is affected by circulating viral genomic variability is currently unknown.

**Objectives:**

To examine whether the vaccine effectiveness of maternal RSVpreF administration in preventing hospitalisation in infants is affected by RSV type or lineage.

**Methods:**

We will conduct whole genome sequencing of RSV positive samples from infants hospitalised with acute lower respiratory tract infection (ALRI) in the 2024-2025 winter season, at multiple hospitals in England and Scotland, to calculate the relative vaccine effectiveness (rVE) of maternal RSVpreF vaccination by virus type (RSV-A and RSV-B). rVE will be calculated using a case control logistic regression with adjustment by infant age and admission date; sex, socioeconomic status and hospital location will be included as potential confounders if they are associated with a >3% change in rVE. We will also perform a test negative design to examine the VE for RSV-A and RSV-B separately, using RSV-negative controls from hospitals where cases were admitted. Finally, we will compare viral lineages in vaccinated versus unvaccinated infants.

**Results and conclusions:**

Our study will identify whether currently circulating RSV genomic variability impacts on rVE. Confirmation of the null hypothesis - that there is no impact of viral genomic variability on rVE – will provide reassurance to policymakers and public health bodies as RSVpreF is rolled out globally. Conversely, an association between RSV type or lineage and decreased vaccine effectiveness will highlight the need for the enhanced comprehensive national and global molecular surveillance of RSV.

## Plain language summary

**Research overview:**

Respiratory syncytial virus, often shortened to be called RSV, is a common virus that can cause severe infections in children. In babies, RSV is more likely to cause severe disease, and RSV disease is a common cause of hospital admission in this group. Recently, an effective vaccine against RSV, given to pregnant women to protect their babies after birth, has been introduced in some countries, including England and Scotland. There are two types of RSV, called RSV-A and RSV-B, which circulate in communities every year; whether the maternal vaccine antibodies transferred to the women’s infants are equally protective against both types isn’t known. We will conduct a study to determine whether the maternal vaccine is equally effective against RSV-A and RSV-B.

**Why is it important?:**

The World Health Organization has recommended that all babies should be protected against RSV infection, and maternal RSV vaccination is likely to be introduced in many countries across the world. If the vaccine is less effective against a particular RSV type, it will be important to monitor the circulation of that RSV type, and consider changing the vaccination so that it is more effective.

**Key takeaway:**

RSV infection causes a significant global burden of disease in babies, which could be reduced by maternal vaccination. Our study will find out whether the type of RSV that a baby is infected with makes it more or less likely for the maternal vaccine to work.

## Background

Respiratory syncytial virus (RSV) is an RNA virus with two antigenic types, RSV-A and RSV-B, that causes yearly seasonal epidemics in countries with temperate climates ^1^. In the United Kingdom in a typical year, RSV cases in infants start to rise in October, peak in December, and then fall ^2^; RSV seasonality is similar in England ^3^ and Scotland ^4^.

In the late summer of 2024, the United Kingdom, following the United States and Argentina ^5^ introduced maternal RSV vaccination, using a stabilised F (fusion) protein (“RSVpreF”; Abrysvo), into its routine immunisation schedule, with vaccination recommended as soon as possible after a gestation of 28 weeks ^6^. At the start of the programme, which is year-round, the vaccine was offered to all pregnant women at a gestation of 28 weeks or more, with women eligible for vaccination until delivery ^7^.

Estimates of maternal RSV vaccine effectiveness are now available from Argentina ^8^ and the UK ^9^ but whether vaccine effectiveness differs by RSV type remains unclear. The final analysis of the pivotal phase 3 MATISSE trial for RSVpreF ^10^ showed robust protection for RSV-B as an exploratory endpoint. However, this analysis was limited by the relatively small number of typed RSV samples for each group (25 severe RSV-A cases, 61 severe RSV-B cases) and it was not possible to assess protection against RSV-A cases owing to greater uncertainty and wide, overlapping confidence intervals.

RSV genomic variability has been shown to affect the binding of the monoclonal antibodies palivizumab ^11^, suptavumab ^12^ and nirsevimab ^13^, and mutations in the SARS-CoV-2 Spike protein (equivalent to the RSV F-protein) were also shown to affect the ability of neutralising, vaccine induced antibodies to bind to new variants of the virus ^14^. Therefore, there is the theoretical possibility that either existing or newly emerging circulating RSV genomic variability, could impact on the effectiveness of a vaccine that depends on passive, antibody mediated—albeit polyclonal—immunity.

The World Health Organization has recently recommended that interventions to prevent RSV disease (infant passive immunisation or maternal vaccination) should be introduced to protect all infants globally ^15^. Therefore, the number of vaccinated mothers is likely to increase markedly in the next few years. In this analysis, we will examine whether RSV type or lineage within each type is associated with reduced maternal RSVpreF vaccine effectiveness in infants.

## Study protocol

This protocol is structured in keeping with the principles of the STROBE statement.

### Study design

We will conduct a case control study, with samples positive for each RSV type acting as cases/controls respectively, to assess the relative effectiveness of maternal RSVpreF vaccination against hospitalisation for RSV-associated acute lower respiratory tract infection (ALRI) amongst infants born to vaccine-eligible pregnant mothers. We will compare RSV types using a direct comparison to give a relative vaccine effectiveness (rVE). Sample size calculations were based on preliminary data from the BronchStop study ^9^, looking at coverage in the group of mother/infant pairs where the mother was vaccinated >14 days (“fully vaccinated”, see “Exposure” section below) prior to delivery ( Table 1).

**Table 1.** Preliminary results from BronchStop maternal vaccine effectiveness study ^9^.

	Vaccinated	Total	Proportion vaccinated
**cases**	46	388	12%
**controls**	44	129	34%

Table 2 shows the estimated sample size needed for 80% power, with 95% confidence intervals, to detect the difference in effectiveness by type (calculations performed in GLIM4 ^16^ and Microsoft Excel) To detect a difference of 25% between VE for RSV-A and RSV-B, assuming a 2:1 ratio of RSV-A to RSV-B cases, and vaccine coverage of 34%, an estimated 425 RSV positive samples would need to be sequenced (highlighted row in Table 2), which was compatible with sampling from the participating schemes (HARISS/BronchStop/PHS surveillance, for details on these see below).

**Table 2.** Sample size calculations for relative VE (rVE).

				95%CI FOR rVE	N TO DETECT A DIFFERENCE (80% POWER)	
VE1	VE2	rVE (grp2: grp1)	Proportion vaccinated in grp 1	grp1=240, grp2=160	grp1	grp2
70%	70%	0%	12%	-95% to 45%	n/a	n/a
60%	80%	50%	16%	1% to 73%	405	270
**57.5%**	**82.5%**	**59%**	**17%**	**17% to 78%**	**255**	**170**
55%	85%	67%	18%	31% to 83%	175	115
50%	90%	80%	20%	54% to 90%	95	65

Additionally, a test-negative design looking at the VE separately for RSV-A and RSV-B cases, compared with test-negative controls, will be performed as a secondary analysis. For each RSV-A and RSV-B case an RSV-negative control infant, matched by admission date (epidemiological week) and age (in weeks), will be identified.

### Study population

Eligible infants will be born after August 12, 2024 (Scotland) or September 1, 2024 (England) (the same dates as the commencement of RSV maternal vaccination in the two countries) and admitted to hospital. The upper age limit for infants recruited will be 6 months, as per the age cut-off used in the MATISSE study ^17^. Samples will be from infants who have been admitted to hospital, had a positive rRT-PCR test for RSV, and been assigned a primary or secondary diagnosis consistent with an ALRI, which encompasses the likely most common diagnosis of bronchiolitis (J21*; Table 3). To be included, the RSV test must be positive in the 14 days leading up to hospital admission, or 2 days after the day of admission. Samples will come from the BronchStop ^18^ or HARISS ^19^ (England) partnerships, or as part of routine public health surveillance in Scotland. Public Health Scotland will coordinate the sequencing of Scottish samples, and UKHSA will conduct sequencing of samples from across England. Test-negative controls, also with a diagnosis of ALRI but no RSV positive test, will be identified from the hospitals that admitted cases.

**Table 3.** List of ICD-10 codes for inclusion in the study.

Definition	Code sets
Acute lower respiratory tract infection (ALRI)	Any of the following ICD10 codes in any position J12* Viral pneumonia, not elsewhere classified J18* Pneumonia, organism unspecified J20* Acute bronchitis J21* Acute bronchiolitis **J22** Unspecified acute lower respiratory infection
Acute respiratory infection (ARI), for sensitivity analyses	Any of the following ICD10 codes in any position J00* Acute nasopharyngitis [common cold] J01* Acute sinusitis J02* Acute pharyngitis J03* Acute tonsillitis J04* Acute laryngitis and tracheitis J05* Acute obstructive laryngitis [croup] and epiglottitis J06* Acute upper respiratory infections of multiple and unspecified sites J12* Viral pneumonia, not elsewhere classified J18* Pneumonia, organism unspecified J20* Acute bronchitis J21* Acute bronchiolitis **J22** Unspecified acute lower respiratory infection **J96.0** Acute respiratory failure **J96.9** Respiratory failure, unspecified **J98.1** Pulmonary Collapse **R05** Cough **R06.0** Dyspnoea **R06.1** Stridor **R06.2** Wheezing **R06.8** Other and unspecified abnormalities of breathing **P28.8** Other specified respiratory conditions of newborn

### Study period

Analysis will be limited to samples collected 6 weeks after the introduction of each vaccine programme: September 23, 2024 (6 weeks after August 12, 2024) in Scotland, and October 13, 2024 (6 weeks after September 1, 2024) in England. The end date for eligible samples will be April 31, 2025, by which point RSV incidence had fallen to baseline rates in national surveillance in England ^3^ and Scotland ^4^. The rationale for the 6-week run-in period is to allow 4 weeks for the implementation of catch-up maternal vaccination, and a further 2 weeks to allow the transfer of maternal immunity to the fetus ^20^. A fall in the proportion of vaccinated mothers to below 12% over the period of the study would mean that the case control study was no longer powered to examine the primary outcome; these inclusion criteria maximise the likelihood of including the infants of fully vaccinated mothers only (see “Exposure” section below).

### Cases and controls

A case will be an infant hospitalised with RSV-associated ALRI. Cases are defined as a positive PCR test for RSV A or B, and a diagnosis consistent with an ALRI (see Table 3) on hospital admission identified from hospital records. Controls have the same definition as the case, but for the other RSV type (re. RSV A for RSV B). Additionally, a test-negative design looking at the VE for RSV-A and RSV-B cases, compared with test-negative controls, will be performed as a secondary analysis. In this analysis RSV test-negative participants will be identified from admissions to the same hospital as RSV-positive cases, matching on admission date and age at admission.

### Exposure

The treatment exposure will be maternal RSVpreF receipt status prior to birth amongst both case and control patients. Vaccination status:

- Fully vaccinated: An infant will be considered fully vaccinated if the birth mother had received a dose of the RSV vaccine a least 14 days prior to giving birth.
- Partially vaccinated: RSV vaccine received within 14 days of giving birth; these samples will not be included in the analysis.
- Unvaccinated: No RSV vaccine received during the pregnancy

### Covariables

For the infant, data will be collected on date of birth, sex, location of hospital admission, recruitment scheme (HARISS/BronchStop/PHS) and date of hospital admission and discharge. For the infants’ mother, linked data will be collected on maternal RSVpreF vaccination status (yes/no), date of vaccination if given, date of delivery, gestation at delivery, Index of Multiple Deprivation (IMD; England) and Scottish IMD (SIMD), age and ethnicity, and immunosuppressed status. This could be important in the ability to transfer transplacental antibodies, and these mother/infant pairs may need to be excluded from the analysis. Immunosuppressed mothers will be identified by selecting cohorts eligible for COVID-19 Spring vaccinations as defined in tables 3 and 4 of the COVID-19 chapter of the Green Book ^21^.

### Sequencing

Cycle threshold (Ct) values for samples will be calculated using rRT-PCR prior to sequencing. Sequencing of RSV positive samples will be undertaken using the Talts *et al.* protocol ^22^ at UKHSA, and by either the Dong *et al.* ^23^ or Maloney *et al.* ^24^ protocols in Scotland. Sequences will be allocated to a lineage using the Goya *et al.* classification ^25^.

### Datasets

RSV positive samples will be provided by the HARISS and BronchStop studies (England), and routine public health surveillance in Scotland. For all participants in England, the infant unique patient identifier (NHS number) will be linked to the Maternity Services Dataset (MSDS) to gather complete information on date of birth, gestational age at birth and identify the mother. For the test-negative design, infants matched by age (in weeks) and admission date (epidemiological week) with a negative RSV rRT-PCR test will be identified from the same hospitals cases were admitted to. In England, the NHS numbers of participant’s mothers will be linked to retrieve their RSV vaccine status and other demographic information such as age, IMD, ethnicity, geographical location (IIS) and severely immunosuppressed status (CaaS). For Scotland, the Scottish Linked Pregnancy and Baby Dataset (SLiPBD) will be accessed, using the infant unique Community Health Index (CHI) number, to identify the same information.

### Statistical analysis

First, we will conduct a descriptive analysis reporting all covariables, exposure and demographic information from the mother by RSV-A/RSV-B status of the infant, and RSVpreF vaccination status of the mother. We will compare RSV Ct values for unvaccinated versus fully vaccinated infants, comparing the mean Ct values in each group using a two-sample Students t-test. We will also compare RSV type distributions for the unvaccinated versus fully vaccinated cohort, using a Fisher’s exact test to look for evidence of over/under-representation of RSV-A and RSV-B. We will use a Wilcoxon rank sum test to look for over/under-representation of different RSV lineages samples from the fully vaccinated vs unvaccinated infants.

The maternal RSVpreF vaccine effectiveness against RSV-associated hospitalisation in infants for RSV-A vs RSV-B will be estimated using logistic regression. This will be calculated using the following equation: relative vaccine effectiveness = 100% x (1- incidence rate ratio [IRR]), where IRR denotes the incidence rate ratio for one group (RSV-A) versus the other (RSV-B).

The analysis will adjust for the potential confounders of age in months and admission date (as a spline). The following variables will be included if they lead to a change in the VE of more than 3%: preterm birth, sex, maternal ethnicity, hospital location (both individual site and country) and recruitment scheme. Protection is known to be higher for fully vaccinated infants ^9^; therefore, rVE will be calculated only including fully vaccinated and unvaccinated infants, as per the sample size calculations in Table 1.

The VE for the test-negative design will be calculated using the following equation: effectiveness = 100% x (1- adjusted odds ratio). The analysis will adjust for potential confounders, as outlined above. We will conduct a sensitivity analysis comparing the rVE/VE for protection against lower respiratory tract infection to that for any acute respiratory infection ( Table 3).

## Ethical issues

The BronchStop study was submitted for Integrated Research Application System (IRAS) approval with University Hospitals of Leicester NHS Trust as the Study Sponsor, IRAS ID 297802, and received a favourable opinion from the Research Ethics Committee on August 8, 2024, including for the collection of data and virology samples without the requirement for individual informed patient consent. HARISS surveillance is undertaken under Regulation 3 of The Health Service (Control of Patient Information) Regulations 2002 (UK legislation) which allows for collection of data for public health purposes without the requirement for individual informed patient consent. The UK Health Security Agency (UKHSA) Caldicott Advisory Panel and Research Ethics and Governance Group considered HARISS epidemiological surveillance and granted approval for the surveillance to take place, including for the collection and processing of virology samples. Permissions for data linkage in England relating to RSV vaccine programme surveillance and evaluation are undertaken under Regulation 3 of The Health Service (Control of Patient Information) Regulations 2002 to collect confidential patient information. Permissions for data linkage within Public Health Scotland are covered within the Public Health Data (Infectious Respiratory Diseases) (Scotland) Directions 2024, and by the PHS Data Protection Impact Assessment (DPIA) DP24250011. In summary, for all three sources of samples (BronchStop, HARISS and PHS) informed consent for data collection and viral sample processing was waived as per the ethical and public health permissions outlined above, for the purposes of this public health analysis.

## Data input, storage and management

Consented data for the BronchStop study for samples from included English infants has been entered using the validated online data entry software REDCap. This software (REDCap) is hosted on the University Hospitals Bristol and Weston NHS Foundation Trust (UHBW) secure server, accessible on the Health and Social Care Network (HSCN) that is managed by NHS Digital. Data for the included infants for HARISS was collected at participating sites and shared as per the HARISS protocol using encrypted, password-protected line lists. Infant date of birth and hospital number from BronchStop and HARISS will be provided to UKHSA for linkage to the maternal dataset. Data on samples from infants admitted to hospital in Scotland is shared by NHS Health Boards with Public Health Scotland for linkage with the SLiPB dataset.

## Dissemination of results

Initial rVE estimates will be shared within UKHSA and Public Health Scotland. They will be uploaded to a preprint server (e.g. medRxiv or SSRN) and submitted for publication in a peer-reviewed journal. Results will also be disseminated via national reports by the public health agencies.

## Conclusion

The number of infants who benefit from maternal RSV vaccination is likely to rise soon. An understanding of whether RSV type potentially affects vaccine effectiveness in infants will be of interest to clinicians, public health officials and policy makers globally. A null result showing no evidence of RSV type on vaccine effectiveness will reassure stakeholders and act as a baseline for future studies; conversely, an association between RSV type or lineage and decreased vaccine effectiveness will highlight the need for the ongoing comprehensive national and global molecular surveillance of RSV.

## Data Availability

No data are associated with this article. Data used to make the sample size calculations was taken from preliminary results from the BronchStop study ^9^.
