# Supplementary material for: Study protocol: Effectiveness of the maternal RSVpreF vaccine by virus type
Source: Wellcome Open Res. 2025 Dec 5;10:664. [Version 1] doi: 10.12688/wellcomeopenres.24371.1 (PMC12770888; doi:10.12688/wellcomeopenres.24371.1)
Supplement: Peer review history

# Reviewer report: Reviewer response for version 1

**DOI:** 10.21956/wellcomeopenres.26872.r141900

**Published:** 2026-01-05

**Authors:** Michael G Ison

**Affiliations:** 1 National Institute of Health, Rockville, MD, USA

This outlines a study protocol to assess the vaccine effectiveness of maternal RSV vaccination.  Standard case-control methods and WGS will be utilized.  The information will inform VE and may provide insight into drift if there are genetic changes determined throughout the study.  Sample size is justified.  Ethical issues and data management and dissemination are clearly outlined.

Is the study design appropriate for the research question?

Yes

Is the rationale for, and objectives of, the study clearly described?

Yes

Are sufficient details of the methods provided to allow replication by others?

Yes

Are the datasets clearly presented in a useable and accessible format?

Yes

Reviewer Expertise:

respiratory viral infections

I confirm that I have read this submission and believe that I have an appropriate level of expertise to confirm that it is of an acceptable scientific standard.

# Reviewer report: Reviewer response for version 1

**DOI:** 10.21956/wellcomeopenres.26872.r141899

**Published:** 2025-12-29

**Authors:** Ram Hari Chapagain

**Affiliations:** 1 Kanti Children’s Hospital, Kathmandu, Nepal

RSV is burining issues and there is possiblity of circulting different strains in different time frame and geographical areas.

 It is necessary to know the circulating strains for the better action of the intervention we are using .

 This study is helps to find out the implementing vaccine is useful to control the RSV or not and help  decision maker to plan for the coming year.

Is the study design appropriate for the research question?

Yes

Is the rationale for, and objectives of, the study clearly described?

Yes

Are sufficient details of the methods provided to allow replication by others?

Yes

Are the datasets clearly presented in a useable and accessible format?

Not applicable

Reviewer Expertise:

infectioous disease includign RSV

I confirm that I have read this submission and believe that I have an appropriate level of expertise to confirm that it is of an acceptable scientific standard.
